# Correlation of thyroid stimulating hormone receptor mRNA expression levels in peripheral blood with undesirable clinicopathological features in papillary thyroid carcinoma patients

**DOI:** 10.18632/oncotarget.18273

**Published:** 2017-05-26

**Authors:** Riming Liu, Shaolong Hao, Hua Zhang, Jihong Ma, Xincheng Liu, Jie Xu, Xin Liu, Jinyao Ning, Yan Sun, Lixin Jiang, Guojun Li, Xicheng Song, Haitao Zheng

**Affiliations:** ^1^ Department of Laboratory of Molecular Biology, The Affiliated Yantai Yuhuangding Hospital of Qingdao University, Yantai, Shandong Province, China; ^2^ Department of Thyroid Surgery, The Affiliated Yantai Yuhuangding Hospital of Qingdao University, Yantai, Shandong Province, China; ^3^ Department of Otolaryngology-Head and Neck Surgery, The Affiliated Yantai Yuhuangding Hospital of Qingdao University, Yantai, Shandong Province, China; ^4^ Department of Central Laboratory, The Affiliated Yantai Yuhuangding Hospital of Qingdao University, Yantai, Shandong Province, China; ^5^ Department of Head and Neck Surgery, The University of Texas MD Anderson Cancer Center, Houston, Texas, USA; ^6^ Department of Epidemiology, The University of Texas MD Anderson Cancer Center, Houston, Texas, USA

**Keywords:** papillary thyroid carcinoma, peripheral blood, TSHR mRNA, noninvasive detection

## Abstract

To determine the extent to which thyroid stimulating hormone receptor (TSHR) mRNA in peripheral blood (PB) has diagnostic value for papillary thyroid carcinoma (PTC). We obtained pre- and postoperative PB samples from 104 thyroid disease patients and collected 11 healthy volunteers’ PB samples twice apiece at different times. We used reverse transcription polymerase chain reaction (RT-PCR) to quantify TSHR mRNA expression levels in the samples. *T-test* and chi-square test were used to compare quantitative data and rates. The mean preoperative PB TSHR mRNA expression level of the PTC patients was significantly higher than that of the healthy volunteers. However, on the postoperative day 1, PB TSHR mRNA level of PTC patients significantly decreased but not for healthy controls. Preoperative PB TSHR mRNA expression levels were significantly associated with patient age, capsular invasion status, lymph node metastasis status, and *BRAF*^V600E^ mutation status (*P* < 0.05) but not gender, tumor size, number of cancer foci, or Hashimoto thyroiditis status. Preoperative assessment of the PB TSHR mRNA expression level combined with ultrasonography of the thyroid had better accuracy in the diagnosis of PTC than either method alone did. Moreover, TSHR mRNA expression significantly affected recurrence of PTC patients. Our findings suggest that PB TSHR mRNA expression level is a promising novel biomarker for the early detection, diagnosis, and treatment of PTC. It may serve as a noninvasive means of PTC detection and a prognostic biomarker of residual tumor and help guide further treatment.

## INTRODUCTION

The incidence of papillary thyroid carcinoma (PTC), the most common endocrine malignancy, has risen rapidly in recent decades owing to widespread environmental and dietary changes and an increasing rate of thyroid papillary microcarcinoma (PTMC) [1]. At present, the preoperative diagnosis of PTC is made primarily with thyroid ultrasonography and/or fine needle aspiration biopsy (FNAB) cytology. Both methods have limitations in the diagnosis of PTC. Although improvements in ultrasonography have increased the rate of accurate diagnosis of patients with thyroid disease, some cases are still difficult to identify with thyroid ultrasonography alone, leading to misdiagnoses that have negatively influenced patients’ prognosis and treatment. FNAB cytology is invasive, and its rate of clinical application in many hospitals in China had been considerably low until recently; in addition, FNAB cytology has false positive and false negative rates of about 5% each, which have contributed to misdiagnoses in 10%–30% of suspected PTC cases [2]. Therefore, a new test for the diagnosis of PTC is necessary.

One pathway that plays an important role in PTC development and progression is the mitogen-activated protein kinase (MAPK) signaling pathway. Excessive activation of the MAPK signaling pathway, which increases the risk of PTC development and progression, may be caused by mutations in the B-Raf proto-oncogene, serine/threonine kinase gene, *BRAF*. *BRAF* mutations are the most common gene mutations in PTC; in particular, previous studies have suggested that more than 50% of thyroid cancers have the *BRAF*^V600E^ mutation [3], which is associated with lymph node metastasis, extrathyroidal invasion, and advanced disease stage [4–6]. Therefore, BRAF gene mutations may be associated with the high invasiveness of PTC. Peripheral blood (PB) thyroid stimulating hormone receptor (TSHR) mRNA is one of circulating tumor markers, while it remains unclear if it is affected by BRAF gene mutations. Some studies suggest that BRAF gene mutations may be related with methylation abnormality of TSHR gene, thus affecting the expression of TSHR mRNA and TSHR protein in PTC tissues [7].

In the past 3 decades, although some tumor markers were used in diagnosis and monitoring of thyroid cancer, there were many problems and difficulties in monitoring the development of thyroid cancer [8–11]. TSHR is a transmembrane protein, which exists in the thyroid follicular epithelial cells, and belongs to the G protein-coupled receptor. It is involved in regulation of growth and differentiation of thyroid follicular epithelial cells in combination with TSH. In 1988, Ringel et al. [12] used anti–TSHR antibodies and immunomagnetic separation technology to detect circulating tumor cells (CTCs) in thyroid cancer patients. However, the method Ringel used had low sensitivity and specificity and was difficult to apply clinically, due to few CTCs exist in PB of patients with PTC, decreased expression in PTC cell membrane and the restrictiveness of the enrichment technology of CTCs. In this study, we applied the real-time reverse transcription polymerase chain reaction (RT-PCR) techniques to detect the PTC patients’ PB TSHR mRNA. The PB TSHR mRNA in PTC patients was more easily assessable and had a higher level than that in CTCs. Furthermore, compared with enrichment techniques of CTCs, RT-PCR technique was more mature and easier for use. The purpose of the present study was to assess the diagnostic value of preoperative PB TSHR mRNA expression levels and investigate the effect of the *BRAF*^V600E^ mutation on the expression level of PB TSHR mRNA in PTC patients.

## RESULTS

The 115 participants we recruited included 80 women and 35 men. The participants’ mean age was 44.77 ± 13.39 years (range, 22–77 years). Of the 104 patients with pathologically confirmed thyroid disease, 70 had PTC, The preceding information is given below and in Table 1; and 34 had BTD (22 with nodular goiter; 7 with follicular cell neoplasia, and 5 with HT). All PTC patients underwent unilateral lobectomy plus isthmectomy and unilateral central cervical lymph node dissection. All BTD patients underwent partial thyroidectomy or subtotal thyroidectomy.

**Table 1:** Preoperative thyroid stimulating hormone receptor (TSHR) mRNA expression levels and clinical features of 70 papillary thyroid carcinoma (PTC) patients

**Variable**	**No. of Patients**	**Mean TSHR mRNA Expression Level ± SD**	***P* Value**
Sex			
Male	17	2.34 ± 1.41	0.207
Female	53	2.13 ± 0.11	
Age, years			
< 45	35	1.97 ± 0.92	0.005*
≥ 45	35	2.39 ± 1.39	
Histological type			
PTC	70	2.18 ± 0.14	0.002*
BTD	34	1.46 ± 0.15	
Tumor size, cm			
≤ 1	37	2.01 ± 0.19	0.349
> 1	33	2.32 ± 0.21	
Capsular invasion			
Yes	17	2.69 ± 0.38	0.041*
No	53	2.01 ± 0.14	
Lymph node metastasis			
Yes	44	2.40 ± 0.19	0.033*
No	26	1.81 ± 0.20	
No. of cancer foci			
Single	54	2.08 ± 0.15	0.242
Multiple	16	2.53 ± 0.34	
Hashimoto thyroiditis			
Yes	24	1.82 ± 0.15	0.063
No	46	2.37 ± 0.20	
*BRAF*^V600E^ gene mutation			
Mutant	51	2.38 ± 0.17	0.012*
Wildtype	19	1.63 ± 0.23	

TSHR: thyroid stimulating hormone receptor; PTC: Papillary Thyroid Carcinoma; BTD: benign thyroid disease.

The characteristics of the 70 PTC patients are given in Table 1. Of these 70 patients, 53 (76%) were women, 35 (50%) were ≥ 45 years old, 37 (53%) had PTMC, 17 (24%) had capsular invasion, 44 (63%) had lymph node metastasis, 16 (23%) had multiple cancer foci, 24 (34%) had HT, and 51 (73%) had a *BRAF*^V600E^ mutation.

**Figure 1:**
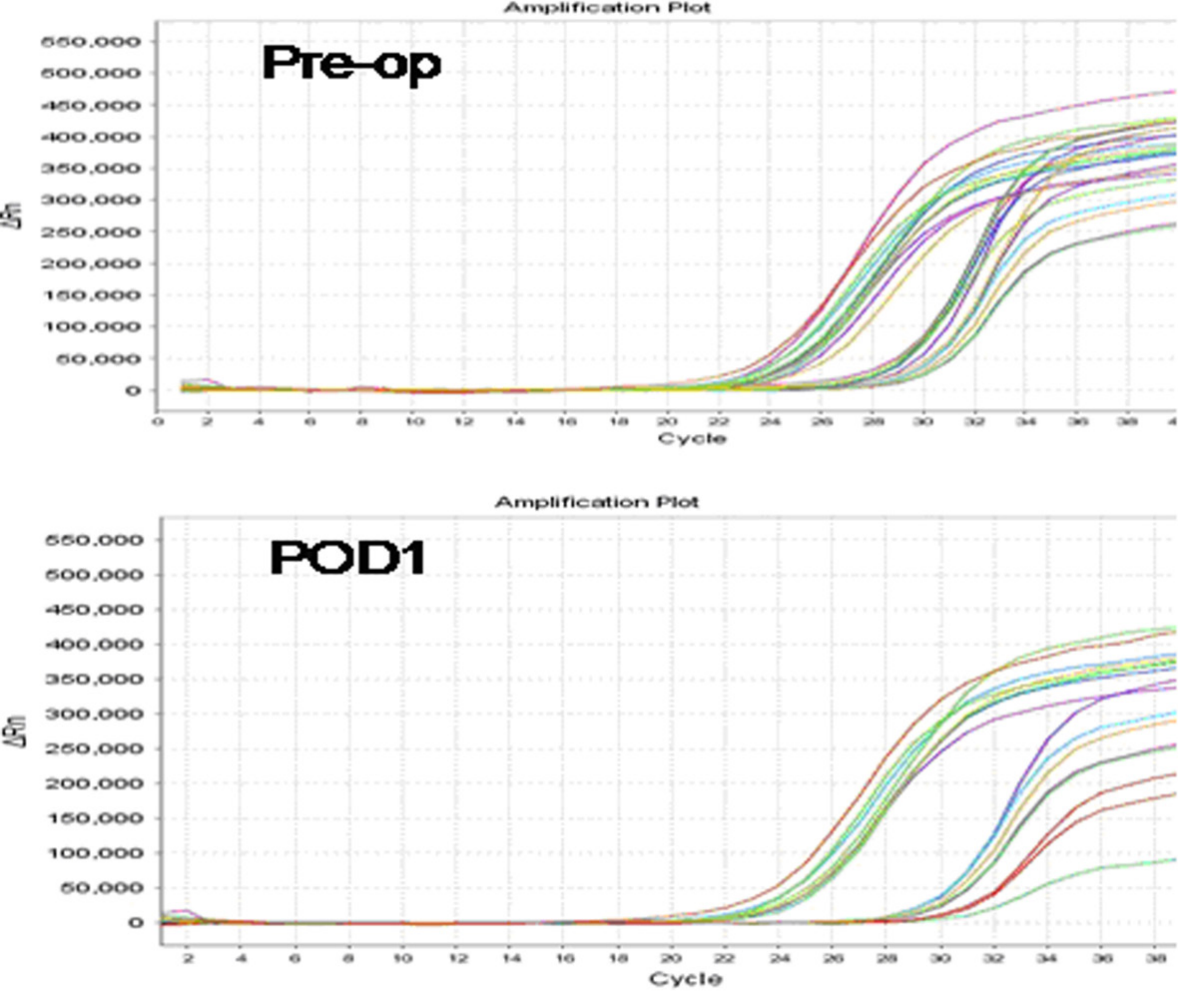
Polymerase chain reaction signaling maps of preoperative thyroid stimulating hormone receptor (TSHR) mRNA expression levels (Pre-op; top) and postoperative day 1 TSHR mRNA expression levels (POD1; bottom) in patients with papillary thyroid carcinoma The sets of curves on the left represent reference mRNA; the sets of curves on the right represent TSHR mRNA.

**Figure 2:**
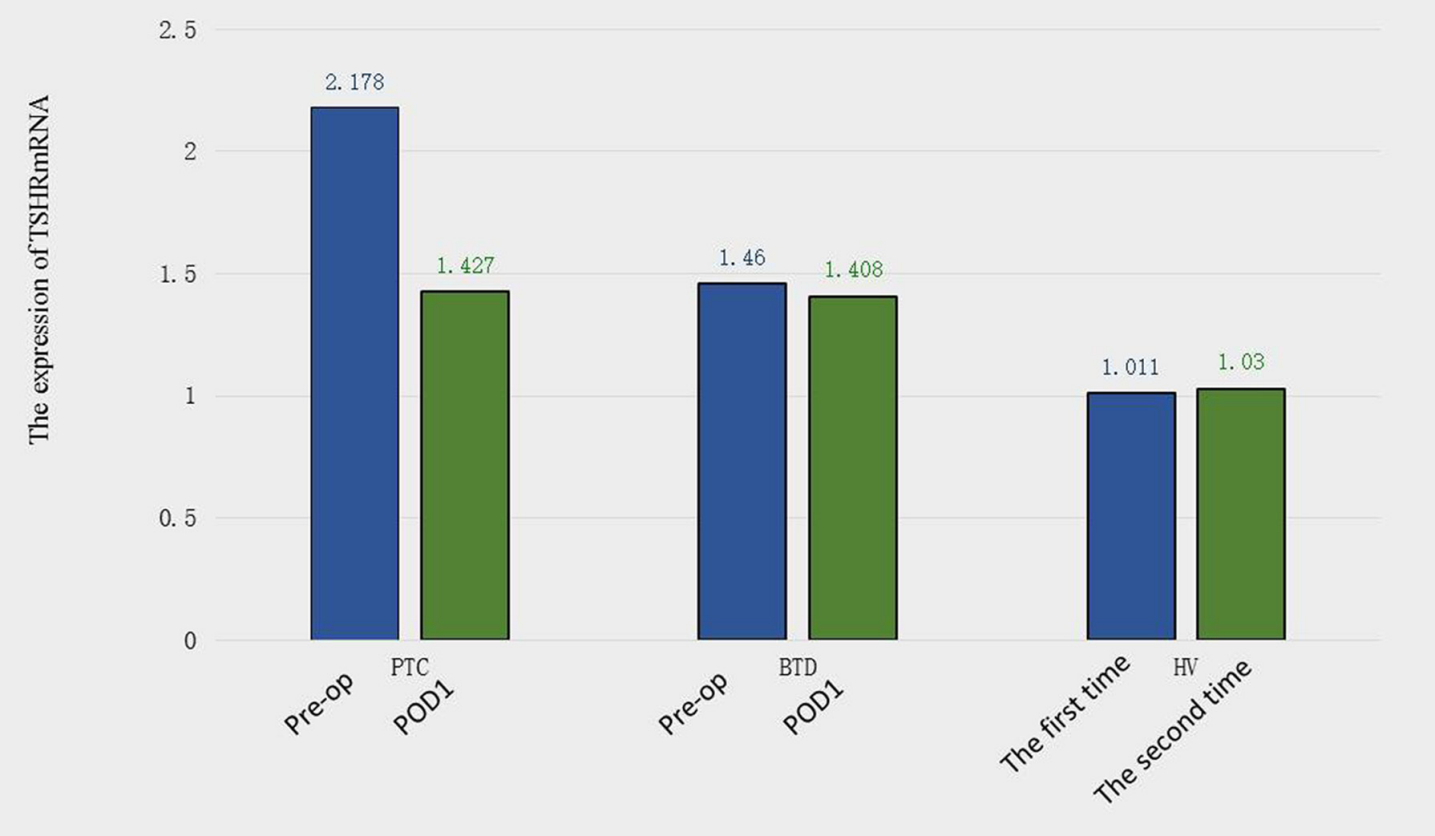
Preoperative (Pre-op) and postoperative day 1 (POD1) thyroid stimulating hormone receptor (TSHR) mRNA expression levels in patients with papillary thyroid carcinoma (PTC) and patients with benign thyroid disease (BTD) Thyroid stimulating hormone receptor (TSHR) mRNA expression levels in healthy volunteers (HV) at different times.

The results of the RT-PCR analysis quantifying pre- and postoperative PB TSHR mRNA expression levels are shown in Figure 1. The mean preoperative PB TSHR mRNA level of the PTC patients (2.178 ± 1.189 ng/ug) was significantly higher than that of the BTD patients (1.460 ± 0.848 ng/ug; *P* = 0.001) and that of the healthy volunteers (1.011 ± 0.171 ng/ug; *P* = 0.001) (Figure 2). The mean preoperative PB TSHR mRNA expression levels of the BTD patients and healthy volunteers did not differ significantly (*P* = 0.217). The mean postoperative TSHR mRNA expression levels of the PTC patients, BTD patients, and healthy volunteers did not differ significantly (Table 2). In addition, for the PTC patients, the mean postoperative PB TSHR mRNA expression level was significantly lower than the mean preoperative TSHR mRNA expression level was (*P* < 0.05).

**Table 2:** Thyroid stimulating hormone receptor (TSHR) mRNA expression on postoperative day 1

**Group**	**No. of Participants**	**Mean TSHR mRNA** **Concentration ± SD, *n*g/*u*g**	***P* Value**
BTD	34	1.41 ± 0.52	> 0.05
PTC	70	1.43 ± 0.76	> 0.05
HV	11	1.03 ± 0.10	> 0.05

TSHR: thyroid stimulating hormone receptor; PTC: Papillary Thyroid Carcinoma; BTD: benign thyroid disease; HV: healthy volunteers.

In this study, our further analysis revealed that PTC patients’ preoperative PB TSHR mRNA expression level was associated with age, histological type, capsular invasion status, lymph node metastasis status, and *BRAF*^V600E^ mutation status (*P* < 0.05) but not gender, tumor size, number of cancer foci, or HT status (*P* > 0.05) (Table 1).

The mean level of preoperative PB TSHR mRNA expression in the PTC patients with wild-type *BRAF* was significantly lower than that in the PTC patients with the *BRAF*^V600E^ mutation, while it was higher than that in the BTD patients. In PTC patients, the preoperative PB TSHR mRNA expression level was positively correlated with lymph node metastasis rates (*r* = 0.387; and *P* = 0.001).

**Figure 3:**
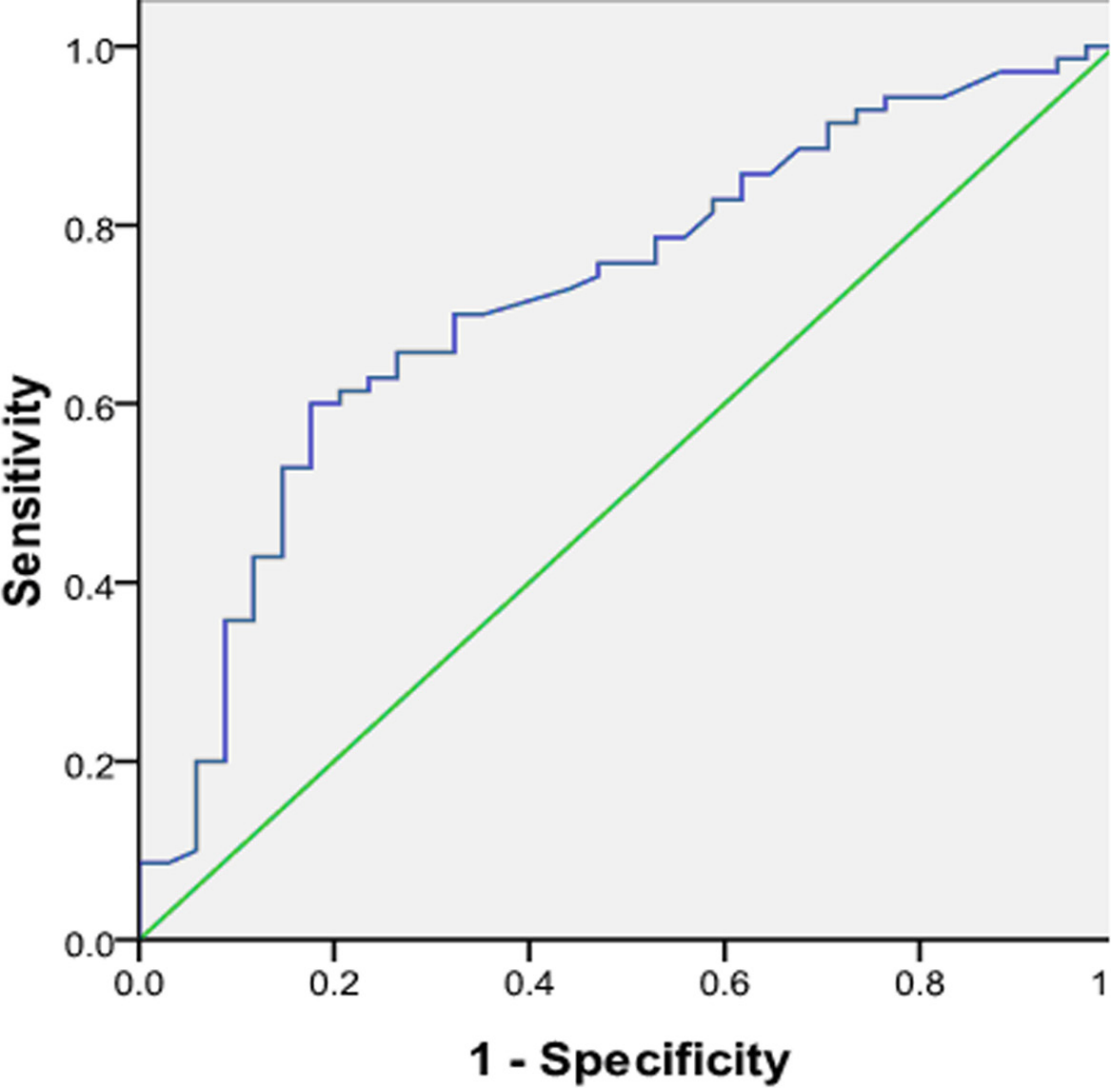
Receiver operating characteristic (ROC) curve demonstrating the diagnostic value of preoperative peripheral blood thyroid stimulating hormone receptor mRNA in papillary thyroid carcinoma patients The area under the ROC curve was 0.722.

A ROC curve was constructed to assess the diagnostic value of preoperative PB TSHR mRNA expression levels for PTC (Figure 3). The area under the ROC curve was 0.722, and the cut-off point of the TSHR mRNA expression level was about 1.70. Using this cut-off value, we found that the TSHR mRNA expression level had an accuracy of 67.3%, sensitivity of 60.0%, specificity of 82.4%, PPV of 87.5%, and NPV of 50.0% in the diagnosis of PTC. We also found that thyroid ultrasonography had an accuracy of 70.2%, sensitivity of 78.6%, specificity of 52.9%, PPV of 77.6%, and NPV of 54.5% in the diagnosis of PTC, indicating that the TSHR mRNA expression level or thyroid ultrasonography alone cannot detect all PTC. However, the TSHR mRNA expression level plus thyroid ultrasonography had an accuracy of 78.8%, sensitivity of 95.7%, specificity of 55.9%, PPV of 77.9%, and NPV of 83.3% in the diagnosis of PTC, indicating that the combination detects PTC with greater precision than either method alone does.

Furthermore, we followed up 70 PTC patients for 18 months and we found 9 patients were lost for follow up. Among 61 PTC patients, there were 6 had disease recurrence and 55 were recurrence-free. The multivariable Cox proportional hazards regression analysis regarding the association between TSHR mRNA expression and risk of recurrence is shown in Table 3. Estimates of association were adjusted for other potential confounders, such as age, gender, stage, treatment, tec. Compared with patients having low expression of TSHR mRNA, the patients with high expression had significantly increased risk of recurrence (HR, 4.1; 95% CI, 1.7–6.4).

**Table 3:** Association between TSHR mRNA expression and recurrence in patients with PTC (*N* = 61)

TSHR mRNA expression	No. of recurrences/No. of patients	HR, 95% CI
Low^†^	3/14	1.0
High	3/47	4.1(1.7–6.4)

^†^Reference group

HR (hazard ratio): adjusted by age, sex, stage, treatment etc.

## DISCUSSION

By interacting with thyroid stimulating hormone, TSHR regulates thyroid function, thyroid cell growth, and thyroid cell differentiation. TSHR may also be expressed in normal thyroid and PTC. But its expression was different between normal thyroid and PTC. In 2002, Gupta et al. posited that TSHR mRNA is a circulating tumor marker for thyroid cancer patients [13]. In 2004, Chinnappa et al. found that thyroid cancer patients had a higher rate of TSHR mRNA expression in the PB than normal thyroid did [14]. Wagner et al. [15], using RT-PCR to detect TSHR mRNA in the PB of patients with benign or malignant thyroid nodules, found that patients with differentiated thyroid carcinoma had a higher level of TSHR mRNA expression in the PB than benign thyroid nodules did.

Our results demonstrate the association between TSHR mRNA expression and recurrence among patients with PTC. We found that patients with the higher TSHR mRNA expression had a significantly higher risk of disease recurrence than did patients with the lower expression. These results may help physicians make decision for individualized treatment. If the association between the TSHR mRNA expression and PTC disease recurrence is confirmed, clinicians could use these expression profiling as a biomarker to identify an important subgroup of patients who are at high risk of recurrence. It is likely that future targeted therapies will be designed (in part) to counteract the effects of significantly higher TSHR mRNA expression as well as individualized within the PTC patients. Furthermore, it may be possible to intensify treatment for patients in whom the higher expression is identified before treatment begins, add adjuvant therapy for those with higher expression identified immediately after treatment, and intensify the workup for treatable recurrent disease or in those who are found to have a high risk of recurrence in follow-up. However, these potential therapeutic options would each have to be tested in clinical trials. It is hoped that the earlier recurrent disease is detected the greater the chances of successfully treating such disease. Ultimately, the findings this study may help improve prognostication, facilitate more selective use of systemic therapy, and hopefully, improve outcomes.

In this study, we confirmed that the preoperative PB TSHR mRNA expression levels of PTC patients are significantly higher than those of BTD patients and healthy volunteers. This suggests that the PB TSHR mRNA expression level could be used as an important biological marker for the preoperative diagnosis of PTC. We also found that for PTC patients, the postoperative PB TSHR mRNA expression levels were significantly lower than preoperative PB TSHR mRNA expression levels; in addition, postoperative PB TSHR mRNA expression levels did not differ significantly among PTC patients, BTD patients, and healthy volunteers (*P* = 0.881). These findings suggest that the postoperative PB TSHR mRNA expression level could be used as an evaluation index, which is consistent with the conclusions of a previous report [16]

Previous studies have shown that preoperative PB TSHR mRNA expression levels are higher in PTMC patients than in BTD patients and healthy individuals. Those studies’ findings suggested that the detection of PB TSHR mRNA was useful in making a preoperative diagnosis of PTMC [17–19]. Similarly, we found that PB TSHR mRNA expression levels were significantly higher in PTMC patients than in BTD patients and healthy volunteers but did not differ significantly between patients with PTMC and those with papillary thyroid non-microcarcinoma. These results indicate that PB TSHR mRNA expression levels could be used for the preoperative diagnosis of PTMC and would not be affected by the size of the cancer foci.

Our study is the first to investigate the influence of the *BRAF*^V600E^ mutation on the preoperative PB TSHR mRNA expression level. In a previous study [20], we found that PTC patients had a BRAFV600E gene mutation rate of about 78%. In the present study, the PB TSHR mRNA expression levels of PTC patients without BRAFV600E mutations were significantly lower than those of PTC patients with BRAFV600E mutations (*P* = 0.012) and significantly higher than those of BTD patients (*P* < 0.01). These results suggest that BRAFV600E mutation status does not affect the accuracy of PB TSHR mRNA expression levels in the diagnosis of PTC. This may be due to the fact that *BRAF*^V600E^ mutations induce abnormal TSHR gene methylation, which negatively affects TSHR mRNA processing. Previous studies suggested that *BRAF*^V600E^ gene mutation was associated with aggressive phenotype of PTC, such as lymph nodes metastasis, extracapsular invasion, and extrathyroid tissue invasion. Thus, we speculate that the hematogenous metastasis more likely occurs when PTC with *BRAF*^V600E^ gene mutation and produces more CTCs, thus leading to elevated levels of PB TSHR mRNA.

Generally, PTC has a favorable prognosis, with a postoperative survival rate up to approximately 90%, while there are still some PTC to have robust invasion. Ries et al. [21] suggested that patients with tumor capsular invasion and/or lateral neck lymph nodes metastasis had poor prognosis. Berber et al. [19] suggested that an increasing PB TSHR mRNA expression level might be a sign of aggressive disease in PTC patients. In our study, an increasing PB TSHR mRNA expression level was related to lymph node metastasis and capsular invasion, which was positively associated with the rate of lymph node metastasis. These findings, which are consistent with those of the study of Berber et al. [19], indicate that the PB TSHR mRNA expression level reflects the aggressiveness of PTC.

PB TSHR mRNA test could be used for diagnosis of PTC, as it might not be affected by nodule-type HT. In recent years, the relationship between HT, a common thyroid autoimmune disease, and thyroid cancer has became a focus of new research. Gul et al. [22] analyzed the clinical data of 613 patients who underwent thyroid surgery and noted the difficulty of using thyroid ultrasonography to differentiate between nodular HT and malignant thyroid nodules. In the present study, we found that the PB TSHR mRNA expression levels of PTC patients with HT and those of PTC patients without HT did not differ significantly (*P* = 0.063),which suggests that HT does not affect preoperative PB TSHR mRNA expression. Therefore, the detection of preoperative PB TSHR mRNA may be used for the diagnosis of PTC and HT.

We also found that the value of the PB TSHR mRNA expression level in the preoperative diagnosis of PTC increased when it was combined with thyroid ultrasonography. The accuracies of thyroid ultrasonography or PB TSHR mRNA expression level alone in the diagnosis of PTC were 70.2% and 67.3%, respectively, whereas the accuracy of the two methods combined was substantially higher, at 78.8%. Although FNAB cytology is still the gold standard for the preoperative diagnosis of PTC, its accuracy can be affected by puncture sites, tumor size, an insufficient sample size, and overlapping factors of tumor cells morphology [23–26]. In addition, because the accuracy of FNAB cytology is closely related to the experience of the pathologist, the diagnosis would be largely subjective, resulting in many false positives and false negatives. Recently, Ren et al. [27] found that the addition of the PB TSHR mRNA expression level significantly improved the sensitivity and specificity of FNAB cytology in the preoperative diagnosis of PTC. This conclusion is consistent with previous research from Wangner et al. [15] and Chia et al. [16].

At present, the diagnosis of PTC relies mainly on thyroid ultrasound and FNAB. Thyroid ultrasound has many limits when thyroid nodules are small in size, locate in dorsal aspect of thyroid, and are associated with nodule-type HT. FNAB belongs to the invasive examination and may also have some limits. For example, when the diameter of thyroid nodule was less than 5 mm, the location of thyroid nodule was close to important blood vessels and/or recurrent laryngeal nerve. The test of the PB TSHR mRNA expression level is a noninvasive examination method. A high PB TSHR mRNA expression level could indicate capsular invasion, lymph node metastasis, and/or *BRAF*^V600E^ mutations. The presence of HT would not affect TSHR mRNA expression level used to make a PTC diagnosis. However, this study has relatively sample sizes and without follow up data of study subjects. Therefore, we didn’t perform the analysis on associations between PB TSHR mRNA and PTC patients’ prognosis, and such studies on associations could be one of our future research focuses.

In conclusion, our findings suggest that the PB TSHR mRNA expression level can be used to help make a preoperative diagnosis of PTC. Our findings also suggest that the preoperative PB TSHR mRNA expression level can supplement thyroid ultrasonography in the evaluation of patients with suspected PTC. In addition, the postoperative PB TSHR mRNA expression level might be used to assess PTC patients for residual tumor and guide postoperative treatment. Maybe the PB TSHR mRNA expression levels is an effect marker for predicting prognosis, evaluating surgical efficacy, planning postoperative follow-up, and monitoring for recurrence in PTC patients in future. Large and prospective studies and long term follow-up are needed to validate our results.

## MATERIALS AND METHODS

### Study participants

We recruited 115 participants (104 previously untreated patients with pathologically confirmed thyroid disease and 11 healthy volunteers) seen in the Department of Thyroid Surgery at Yantai Yuhuangding Hospital of Qingdao University from April 2015 to December 2015. Some patients were excluded if they 1) had non-papillary thyroid carcinoma; 2) had other system malignant tumors or severe system disease; 3) had abnormal PB leukocyte count; 4) had past history of thyroid disease; and 5) had underwent TSH inhibition or Iodine 131 treatment. During that same period, the controls (11 healthy volunteers) had been selected from healthy visitors who had accompanied cancer patients to outpatient clinics at Yuhuangding Hospital but who were genetically unrelated to the patients. The inclusion criteria of healthy volunteers (HV) were as following, if 1) the participants had no possibility of benign and malignant thyroid nodules by B-ultrasonic examination; 2) the thyroid function was normal; 3) the subjects had no history of benign and malignant thyroid diseases; and 4) the subjects had no history of other system malignancies (lung cancer, colon cancer, prostate cancer, etc). We collected the PB samples of HV twice at different time points. We used the average values as the standard of the PB TSHR mRNA in PTC and BTD patients. The study was approved by the hospital’s ethics committee, and all 115 participants provided written informed consent to be included in the study.

Each participant’s gender, age, pathologic disease type, tumor size, capsular invasion status, lymph node metastasis status, number of cancer foci, Hashimoto thyroiditis (HT) status, and *BRAF*^V600E^ gene mutation status were recorded. Ultrasonography and common laboratory tests were obtained for all 115 participants before surgery. Patients with pathologically confirmed thyroid disease were diagnosed with PTC or benign thyroid disease (BTD) on the basis of pathological analysis of formalin-fixed, paraffin-embedded tissue samples obtained during diagnostic or therapeutic surgery. The tumor ≤ 1 cm along the largest diameter is defined as PTMC. A receiver operating characteristic (ROC) curve was constructed to assess the diagnostic value (Such as accuracy, sensitivity, and specificity, positive predictive value (PPV) and negative predictive value (NPV)) of preoperative PB TSHR mRNA expression levels and ultrasonography for PTC. And the diagnostic value of the combination of ultrasonography and TSHR mRNA expression level was compared with that of either method alone.

### Sample collection

PB samples (8 ml each) were collected from patients in ethylenediaminetetraacetic acid–containing tubes on the day of surgery preoperatively and in the morning of the day after surgery under fasting conditions. The healthy volunteers were collected PB samples (8 ml each) twice apiece at the same times. The samples were then stored at 4°C.

### TSHR mRNA measurement

Total RNA in blood samples was extracted by TRIzol within 2–4 h, stored at –80°C, and subjected to real-time fluorescent quantitative PCR (probe method) detection within 1 week. We used RT-PCR to measure TSHR mRNA levels in the PB samples. Total RNA was extracted from the cellular fraction of the PB sample without serum or erythrocytes. Amplification of TSHR mRNA was performed using specifically designed primers (upstream primer, 5′-GCTTTTGAAGGGACATGCAATGAA-3′; downstream primer, 5′-AAGGGCCAGTGACACTGGTTTGAGA-3′). We used Taqman real time PCR technique by ABI7500 (Thermo Fisher Scientific, Waltham, MA USA). PCR included a total of 40 cycles, each at 42°C for 30 min, 94°C for 5 min, 94°C for 15 sec, and 60°C for 60 sec. For each sample, TSHR mRNA was quantified by normalization with glyceraldehyte-3-phosphate dehydrogenase mRNA in a separate reaction tube within the same run. The Ct value of the target gene (TSHR) and internal gene (GAPDH) was obtained by machine (ABI 7500 Real Time PCR System, ABI, USA). The relative expression level of TSHR mRNA was measured using the 2^-∆∆CT^ method and reported as the reference equivalent of TSHR mRNA in ng/ug. TSHR mRNA levels > 1.7 ng/ug were interpreted as signifying the presence of thyroid cancer by ROC analysis.

### *BRAF*^V600E^ gene testing

The determination of BRAF^V600E^ gene mutation was performed for all PTC patients by PCR techniques. DNA was extracted using DNA Extraction kit (Promega Corporation, CA, USA) and BRAF gene exon 15 was detected using BRAF mutant gene detection kit (Amoy Diagnostics Co., LTD, China) and the ABI7500 real-time PCR amplifier (Promega Corporation, CA, USA). The primers for amplification of exon 15 of BRAF were as follows: forward (5′-TCATAATGCTTGCTCTGATAGGA-3′) and reverse (5′-GGCCAAAAATTTAATCAGTGGA-3′). All the procedures and analysis were conducted in the biomolecular laboratory of our hospital.

### Statistical analysis

Statistical analyses were performed using the SPSS 17.0 software program. TSHR mRNA expression levels in pre- and postoperative PB samples are given as means ± standard deviations. A *t*-test was used to compare quantitative data between two groups. The chi-square test was used to compare rates. The primary endpoint in this study is recurrence. Time to recurrence will be computed from date of end of treatment to date of last follow-up or date of clinically detectable recurrent cancer. Participants who are recurrence-free or lost to follow-up will be considered censored. Medical record review for follow-up status of all patients was performed under direct supervision of staff head and neck surgeon. Primary tumor subsite, clinical stage, treatment, and vital status were reviewed from medical records as assessed between the initial and final patient contact recorded. The cox proportional hazards regression was applied to estimate the individual hazard ratio (HR) for the recurrence. Statistical significance was set at *P* < 0.05.

## Abbreviations

TSHR: thyroid stimulating hormone receptor
PB: peripheral blood
PTC: papillary thyroid carcinoma
PTMC: papillary thyroid microcarcinoma
RT-PCR: real-time reverse transcriptase polymerase chain reaction
POD: postoperative day
HT: hashimoto thyroiditis
FNAB: fine needle aspiration biopsy
BTD: benign thyroid disease
ROC: receiver operating characteristic
PPV: positive predictive value
NPV: negative predictive value
